# The Advisory Committee on Immunization Practices’ Recommendation for Use of Moderna COVID-19 Vaccine in Adults Aged ≥18 Years and Considerations for Extended Intervals for Administration of Primary Series Doses of mRNA COVID-19 Vaccines — United States, February 2022

**DOI:** 10.15585/mmwr.mm7111a4

**Published:** 2022-03-18

**Authors:** Megan Wallace, Danielle Moulia, Amy E. Blain, Erin K. Ricketts, Faisal S. Minhaj, Ruth Link-Gelles, Kathryn G. Curran, Stephen C. Hadler, Amimah Asif, Monica Godfrey, Elisha Hall, Anthony Fiore, Sarah Meyer, John R. Su, Eric Weintraub, Matthew E. Oster, Tom T. Shimabukuro, Doug Campos-Outcalt, Rebecca L. Morgan, Beth P. Bell, Oliver Brooks, H. Keipp Talbot, Grace M. Lee, Matthew F. Daley, Sara E. Oliver

**Affiliations:** ^1^CDC COVID-19 Emergency Response Team; ^2^Epidemic Intelligence Service, CDC; ^3^College of Medicine, University of Arizona, Phoenix, Arizona; ^4^Department of Health Research Methods, Evidence, and Impact, McMaster University, Hamilton, Ontario; ^5^University of Washington, Seattle, Washington; ^6^Watts Healthcare Corporation, Los Angeles, California; ^7^Vanderbilt University School of Medicine, Nashville, Tennessee; ^8^Stanford University School of Medicine, Stanford, California; ^9^Institute for Health Research, Kaiser Permanente Colorado, Denver, Colorado.

The mRNA-1273 (Moderna) COVID-19 vaccine is a lipid nanoparticle-encapsulated, nucleoside-modified mRNA vaccine encoding the stabilized prefusion spike glycoprotein of SARS-CoV-2, the virus that causes COVID-19. During December 2020, the vaccine was granted Emergency Use Authorization (EUA) by the Food and Drug Administration (FDA), and the Advisory Committee on Immunization Practices (ACIP) issued an interim recommendation for use among persons aged ≥18 years (*1*), which was adopted by CDC. During December 19, 2020–January 30, 2022, approximately 204 million doses of Moderna COVID-19 vaccine were administered in the United States (*2*) as a primary series of 2 intramuscular doses (100 *μ*g [0.5 mL] each) 4 weeks apart. On January 31, 2022, FDA approved a Biologics License Application (BLA) for use of the Moderna COVID-19 vaccine (Spikevax, ModernaTX, Inc.) in persons aged ≥18 years (*3*). On February 4, 2022, the ACIP COVID-19 Vaccines Work Group conclusions regarding recommendations for the use of the Moderna COVID-19 vaccine were presented to ACIP at a public meeting. The Work Group’s deliberations were based on the Evidence to Recommendation (EtR) Framework,* which incorporates the Grading of Recommendations, Assessment, Development and Evaluation (GRADE) approach^†^ to rank evidence quality. In addition to initial clinical trial data, ACIP considered new information gathered in the 12 months since issuance of the interim recommendations, including additional follow-up time in the clinical trial, real-world vaccine effectiveness studies, and postauthorization vaccine safety monitoring. ACIP also considered comparisons of mRNA vaccine effectiveness and safety in real-world settings when first doses were administered 8 weeks apart instead of the original intervals used in clinical trials (3 weeks for BNT162b2 [Pfizer-BioNTech] COVID-19 vaccine and 4 weeks for Moderna COVID-19 vaccine). Based on this evidence, CDC has provided guidance that an 8-week interval might be optimal for some adolescents and adults. The additional information gathered since the issuance of the interim recommendations increased certainty that the benefits of preventing symptomatic and asymptomatic SARS-CoV-2 infection, hospitalization, and death outweigh vaccine-associated risks of the Moderna COVID-19 vaccine. On February 4, 2022, ACIP modified its interim recommendation to a standard recommendation^§^ for use of the fully licensed Moderna COVID-19 vaccine in persons aged ≥18 years.

## Recommendations for Use of Moderna COVID-19 Vaccine

During June 2020–February 2022, ACIP convened 23 public meetings to review data on the epidemiology of COVID-19 and considerations for use of all COVID-19 vaccines, including the Moderna COVID-19 vaccine (*4*). The ACIP COVID-19 Vaccines Work Group, which includes experts in infectious diseases, vaccinology, vaccine safety, public health, and ethics, held meetings each week to review COVID-19 epidemiologic and surveillance data on vaccine efficacy, effectiveness, and safety and implementation considerations. After a systematic review of published and unpublished scientific evidence for benefits and harms^¶^ of Moderna COVID-19 vaccination, the Work Group used a modified GRADE approach to assess the certainty of evidence for outcomes related to the vaccine, rated on a scale of type 1 to type 4 (type 1 = high certainty, type 2 = moderate certainty, type 3 = low certainty, and type 4 = very low certainty). Within the EtR Framework, ACIP considered the importance of COVID-19 as a public health problem, benefits and harms (as informed by the GRADE evidence assessment), patients’ values and preferences, issues of resource use, acceptability to stakeholders, feasibility of implementation, and anticipated impact on health equity. Work Group conclusions regarding the evidence for the Moderna COVID-19 vaccine were presented to ACIP at a public meeting on February 4, 2022.**

The body of scientific evidence for potential benefits and harms of the Moderna COVID-19 vaccine was guided by one large randomized, double-blind, placebo-controlled Phase III clinical trial (*5*,*6*), one Phase II clinical trial (*7*), one small Phase I clinical trial (*8*,*9*), 26 observational vaccine effectiveness studies, and two postauthorization vaccine safety monitoring systems: the Vaccine Adverse Events Reporting System (VAERS) and the Vaccine Safety Datalink (VSD). VAERS is a national passive surveillance vaccine safety monitoring system managed by CDC and FDA. VSD covers nine participating integrated health care organizations serving approximately 12 million persons and identifies possible adverse events after vaccination, using detailed clinical and demographic data available in near real time from electronic medical records. Updated findings from the ongoing Phase III clinical trial were based on 30,420 enrolled participants contributing approximately 11,000 person-years of data, with a median follow-up of 5 months during September 4, 2020–March 26, 2021 (ending with the date placebo recipients were offered crossover to receive study vaccine). Pooled effectiveness estimates were calculated when multiple observational studies reported data on a specific outcome; the study periods for the observational studies included in the pooled estimates ranged from 1 to 10 months (median = 5 months).

The estimated efficacy of the Moderna COVID-19 vaccine in the Phase III clinical trial was based on outcomes that occurred ≥14 days after receipt of the second dose. The demographic characteristics of participants, including age and race (*5*), have remained consistent since initial enrollment. Efficacy in preventing symptomatic, laboratory-confirmed COVID-19 in persons aged ≥18 years without evidence of previous SARS-CoV-2 infection was 92.7% (Table 1). One hospitalization occurred among the vaccinated group and 24 hospitalizations among the placebo group, yielding an estimated vaccine efficacy of 95.9% against COVID-19–associated hospitalization. No COVID-19–associated deaths occurred among study participants in the vaccinated group, and three occurred in the placebo group resulting in a vaccine efficacy of 100% against COVID-19–associated deaths. Efficacy in preventing asymptomatic SARS-CoV-2 infection was 57.4%. Observational data were available for all beneficial outcomes assessed: the pooled vaccine effectiveness estimates were 89.2% for prevention of symptomatic, laboratory-confirmed COVID-19 (11 studies); 94.8% against COVID-19–associated hospitalizations (15 studies), 93.8% against COVID-19–associated death (five studies), and 69.8% against asymptomatic SARS-CoV-2 infection (three studies). Most of the follow-up time occurred before B.1.1.529 (Omicron) became the predominant circulating SARS-CoV-2 variant. From the GRADE evidence assessment, the level of certainty for the benefits of Moderna COVID-19 vaccination among persons aged ≥18 years was type 1 (high certainty) for the prevention of symptomatic SARS-CoV-2 infection, type 1 (high certainty) for the prevention of asymptomatic SARS-CoV-2 infection, type 2 (moderate certainty) for prevention of COVID-19–associated hospitalization, and type 2 (moderate certainty) for the prevention of COVID-19–associated death.

**TABLE 1:** Summary of the certainty of evidence of potential benefits of Moderna COVID-19 vaccination — United States, February 2022

Potential benefit	Clinical trial evidence		Observational evidence		GRADE evidence certainty^†^
	No. of studies	Vaccine efficacy (95% CI)	No. of studies	Pooled vaccine effectiveness* (95% CI)	
Prevention of symptomatic, laboratory-confirmed COVID-19^§^	1	92.7 (90.4–94.4)	11	89.2 (82.0–93.6)	1
Prevention of COVID-19–associated hospitalization^§^	1	95.9 (69.5–99.4)	15	94.8 (93.1–96.1)	2
Prevention of COVID-19–associated death	1	100 (NE–100)	5	93.8 (91.5–95.4)	2
Prevention of asymptomatic SARS-CoV-2 infection	1	57.4 (50.1–63.6)	3	69.8 (60.9–76.7)	1

**Abbreviations**: GRADE = Grading of Recommendations, Assessment, Development and Evaluation; NE = not evaluable.

* Vaccine effectiveness estimates were pooled to provide an overall estimate across studies for the purposes of GRADE review.

^†^ GRADE evidence certainty: 1 = high certainty, 2 = moderate certainty, 3 = low certainty, 4 = very low certainty.

^§^ Considered a critical outcome in GRADE. https://www.cdc.gov/vaccines/acip/recs/grade/about-grade.html

In the Phase III clinical trial, severe local and systemic adverse reactions (i.e., reactogenicity) in the 7 days after vaccination (grade 3 or higher,^††^ defined as adverse reactions interfering with daily activity) were more likely to occur among vaccine recipients (21.3%) than placebo recipients (4.5%) (relative risk = 5.03; 95% CI = 4.65–5.45) (Table 2). Among vaccine recipients, the most common grade 3 symptoms were fatigue, headache, joint pain, muscle pain, and injection-site pain. Overall, reactions categorized as grade 3 or higher were more likely to be reported after the second dose than after the first dose. The frequency of serious adverse events^§§^ was 1.7% among vaccine recipients and 1.9% among placebo recipients. Based on data from VAERS and VSD, two rare but clinically serious adverse events after vaccination were detected: anaphylaxis and myocarditis or myopericarditis.^¶¶^ Based on VSD data, 5.1 cases of anaphylaxis per 1 million doses of Moderna COVID-19 vaccine administered among persons aged ≥18 years were observed (*10*). Myocarditis or pericarditis were more common among vaccine recipients who were younger and male, and occurred more frequently after the second vaccine dose; 65.7 cases per 1 million doses of Moderna COVID-19 vaccine administered were observed from analysis of VSD chart-reviewed myocarditis and myopericarditis cases that met CDC case definitions (*11*) among men aged 18–39 years after dose 2 and occurring within a 0–7-day risk interval after vaccination. Although VAERS data are subject to the limitations of a passive surveillance system,*** the elevated number of observed versus expected myocarditis and myopericarditis cases during the 0–7-day risk interval after receipt of the second Moderna vaccine dose is generally consistent with the findings from VSD. The level of certainty from the GRADE evidence assessment regarding potential harms after vaccination was type 2 (moderate certainty) for serious adverse events and type 1 (high certainty) for reactogenicity. GRADE was last completed for Moderna COVID-19 primary vaccination in December 2020 (*1*); since that time, additional data became available on all prespecified outcomes of interest, resulting in a higher level of certainty in the estimates for the benefit of vaccination in prevention of asymptomatic infection and death (the GRADE evidence profile is available at https://www.cdc.gov/vaccines/acip/recs/grade/bla-covid-19-moderna-vaccine.html). Overall, the benefits for the Moderna COVID-19 vaccine outweigh any observed vaccine-associated risks (Table 1) (Table 2).

**TABLE 2:** Summary of the certainty of evidence of potential harms of Moderna COVID-19 vaccination — United States, February 2022

Characteristic	Clinical trial evidence		Observational evidence		GRADE evidence certainty*
	No. of studies	Relative risk (95% CI)	No. of studies	No. of cases per 1 million doses	
**Potential harms, pooled data **					
**Reactogenicity**	2	5.03 (4.65–5.45)	0	—**^†^**	1
**Serious adverse events^§^**	2	0.92 (0.78–1.08)	0	—^¶^	2
**Potential harms by data source**					
**VSD**					
Anaphylaxis, persons ≥18 yrs	NA	NA	1	5.1**	3
Myocarditis, sex and age group, yrs					
Men, 18–39	NA	NA	1	65.7^††^	3
Women, 18–39	NA	NA	1	6.2^††^	3
**VAERS**					
Myocarditis, sex and age group, yrs					
Men, 18–24	NA	NA	1	40.0^§§^	3
Women, 18–24	NA	NA	1	5.5^§§^	
Men, 25–29	NA	NA	1	18.3^¶¶^	
Women, 25–29	NA	NA	1	5.8^¶¶^	
Men, 30–39	NA	NA	1	8.4***	
Women, 30–39	NA	NA	1	0.6***	

**Abbreviations:** GRADE = Grading of Recommendations, Assessment, Development and Evaluation; NA = not applicable; RR = relative risk; VAERS = Vaccine Adverse Event Reporting System; VSD = Vaccine Safety Datalink.

* GRADE evidence certainty is ranked as follows: 1 = high certainty, 2 = moderate certainty, 3 = low certainty, 4 = very low certainty.

^†^ Observational evidence did not include a measure of reactogenicity.

^§^ Considered a critical outcome in GRADE. https://www.cdc.gov/vaccines/acip/recs/grade/about-grade.html

^¶^ Observational evidence did not include an aggregate measure of serious adverse events. Data on specific serious adverse events identified through postauthorization safety surveillance were reviewed. Increased risk for myocarditis and anaphylaxis were observed in VAERS and VSD.

** Based on VSD chart reviewed cases of anaphylaxis, in persons aged ≥18 years, occurring in a 0–1-day risk interval after vaccination (RR = 5.1; 95% CI = 3.3–7.6).

^††^ Based on VSD chart-reviewed cases of myocarditis and pericarditis that met CDC case definitions among persons aged 18–39 years after dose 2, occurring in a 0–7-day risk interval after vaccination.

^§§^ Based on VAERS chart-reviewed cases of myocarditis that met CDC case definitions among men and women aged 18–24 years, days 0–7 after dose 2.

^¶¶^ Based on VAERS chart-reviewed cases of myocarditis that met CDC case definitions among men and women aged 25–29 years, days 0–7 after dose 2.

*** Based on VAERS chart-reviewed cases of myocarditis that met CDC case definitions among men and women aged 30–39 years, days 0–7 after dose 2.

Data reviewed within the EtR Framework support the use of the Moderna COVID-19 vaccine. The Work Group concluded that COVID-19 remains an important public health problem and that the desirable effects of disease prevention through vaccination with Moderna COVID-19 vaccine in persons aged ≥18 years are large and outweigh the potential harms. With 204 million doses of Moderna COVID-19 vaccine administered to date (*2*), the Work Group determined that the vaccine is acceptable to vaccine providers and that implementation of vaccination is feasible. The Work Group also acknowledged that vaccine-eligible persons aged ≥18 years probably considered the desirable effects of vaccination to be favorable compared with the undesirable effects; however, there is likely important variability in vaccine acceptance within this age group, especially among those who are currently unvaccinated. Despite having recommendations for the Moderna COVID-19 vaccine for >1 year, data indicate vaccine coverage varies by geography, race/ethnicity, sexual orientation, and gender identity (*12*–*14*). Because these disparities remained even after the Pfizer COVID-19 vaccine had received standard authorization, the Work Group concluded that changing from an interim to a standard ACIP recommendation alone for the Moderna COVID-19 vaccine would probably have minimal impact on health equity (the evidence used to inform the EtR is available at https://www.cdc.gov/vaccines/acip/recs/grade/bla-covid-19-moderna-etr.html).

## Interval Between Primary mRNA COVID-19 Vaccination Series Doses

In addition to data presented to guide the recommendation for use of the Moderna COVID-19 vaccine, data were also presented to ACIP regarding the optimal interval between the first and second dose of a Moderna or Pfizer-BioNTech mRNA primary vaccination series. mRNA COVID-19 vaccines are safe and effective at the authorized interval between the first and second doses (4 weeks for Moderna vaccine; 3 weeks for Pfizer-BioNTech vaccine), but an extended interval might be considered for some populations. An elevated risk for myocarditis and myopericarditis among mRNA COVID-19 vaccine recipients has been observed, particularly in adolescent and young adult males (*11*,*15*). Several studies in adolescents and adults have indicated the small risk for myocarditis associated with mRNA COVID-19 vaccines might be reduced (*16*) and peak antibody responses and vaccine effectiveness might be increased (*17*–*20*) with an interval longer than 4 weeks between the 2 primary series doses. In a population-based cohort study in Ontario, Canada, rates of myocarditis among persons aged ≥18 years were lower with an extended interval (>4 to <8 weeks and ≥8 weeks) compared with the shorter interval (3–4 weeks) between the first and second doses of a primary series for both Moderna and Pfizer-BioNTech vaccines (*16*). In several studies, neutralizing antibody titers were higher after an extended interval between doses in a primary mRNA vaccine series (range = 6–14 weeks), compared with a standard interval of 3–4 weeks (*17*–*20*). Vaccine effectiveness against infection and hospitalization was higher with an extended (6–8-week) interval than with a standard (3–4-week) interval (*19*).^†††^ Based on this evidence presented to ACIP, CDC has provided guidance that an 8-week interval might be optimal for some adolescents and adults, especially for males aged 12–39 years. Additional primary series interval considerations are available at https://www.cdc.gov/vaccines/covid-19/clinical-considerations/covid-19-vaccines-us.html.

After a year of use under an FDA-issued EUA and ACIP interim recommendation, the Moderna COVID-19 vaccine received full FDA approval and is recommended by ACIP for use in persons aged ≥18 years in the United States. Spikevax, the trade name of the Moderna COVID-19 vaccine, has the same formulation and can be used interchangeably with the Moderna COVID-19 vaccine used under EUA without presenting any safety or effectiveness concerns. ACIP considered new information beyond what was available at the time of the interim recommendation, including an additional 3 months of follow-up of the Phase III clinical trial participants, 26 observational vaccine effectiveness studies involving large populations of vaccinated persons, and two postauthorization safety monitoring systems with data from millions of vaccinated persons in the United States. The additional information increased certainty that the benefits of Moderna COVID-19 vaccine outweigh vaccine-associated risks. The Moderna COVID-19 vaccine continues to have FDA authorization and interim ACIP recommendations for a booster dose (*21*), as well as an additional dose in persons aged ≥18 years with moderate to severe immunocompromise (*22*). For an mRNA primary series, an 8-week interval between first and second doses might be optimal for some persons aged ≥12 years, especially males aged 12–39 years.

Before vaccination, a fact sheet (*23*) or vaccine information sheet should be provided to recipients. Providers should counsel Moderna COVID-19 vaccine recipients about expected systemic and local reactogenicity. Additional clinical considerations for COVID-19 vaccine administration are available at https://www.cdc.gov/vaccines/covid-19/clinical-considerations/covid-19-vaccines-us.html

## Reporting of Vaccine Adverse Events

Providers are required to report adverse events that occur after receipt of any COVID-19 vaccine to VAERS (*23*) (https://vaers.hhs.gov/index.html or 1–800–822–7967). Any person who administers or receives a COVID-19 vaccine is encouraged to report any clinically significant adverse event, regardless of whether it is clear that a vaccine caused the adverse event. In addition, all COVID-19 vaccine recipients are encouraged to enroll in v-safe, a CDC voluntary smartphone-based online tool that uses text messaging and online surveys to conduct periodic health check-ins after vaccination. CDC’s v-safe (https://www.cdc.gov/vsafe) call center follows up on reports to v-safe that include possible medically significant health events to collect additional information for completion of a VAERS report.

**Summary**

**What is already known about this topic?**

On January 31, 2022, the Food and Drug Administration (FDA) granted full approval to the Moderna COVID-19 vaccine for persons aged ≥18 years.

**What is added by this report?**

On February 4, 2022, after a systematic review of the evidence, the Advisory Committee on Immunization Practices issued a standard recommendation for use of the Moderna COVID-19 vaccine in persons aged ≥18 years. CDC provided guidance that an 8-week interval between primary series doses of mRNA vaccines might be optimal for some persons.

**What are the implications for public health practice?**

Use of the FDA-approved Moderna COVID-19 vaccine is recommended for persons aged ≥18 years; benefits of the prevention of infection and associated hospitalization or death outweigh vaccine-associated risks.

## Footnotes

*
https://www.cdc.gov/vaccines/acip/recs/grade/downloads/acip-evidence-recs-framework.pdf

†
https://www.cdc.gov/vaccines/acip/recs/grade/about-grade.html

§ On February 4, 2022, ACIP voted unanimously in favor of the recommendation for use of Moderna COVID-19 vaccine for persons aged ≥18 years under the FDA BLA.

¶ Evaluated benefits were prevention of symptomatic, laboratory-confirmed COVID-19 and associated hospitalization or death, and asymptomatic SARS-CoV-2 infection. Harms evaluated were serious adverse events and reactogenicity (grade 3 or higher).

**
https://www.cdc.gov/vaccines/acip/meetings/downloads/slides-2022-02-04/07-COVID-Oliver-508.pdf

†† Grade 1 (mild): does not interfere with activity; grade 2 (moderate): some interference with activity; Grade 3 (severe): prevents daily activity; and grade 4: emergency department visit or hospitalization. Some reactogenicity grade categories are symptom specific. https://www.cdc.gov/vaccines/covid-19/info-by-product/moderna/reactogenicity.html

§§ Serious adverse events are defined as any untoward medical occurrence that results in death, is life-threatening, requires inpatient hospitalization or prolongation of existing hospitalization, or results in persistent disability or incapacity; suspected transmission of any infectious agent via a medicinal product; and a medically important event.

¶¶ Myocarditis is an adverse event defined as inflammation of the heart muscle and is called myopericarditis when accompanied by pericarditis, an inflammation of the thin tissue surrounding the heart (the pericardium).

*** Limitations of VAERS are that, as a passive reporting system, there might be bias in reporting, inconsistent data quality and completeness of information, and lack of a direct comparison group. The VAERS system was not designed to assess causality, and therefore VAERS data generally cannot determine if a causal association between an adverse event and a vaccine exists.

†††
https://www.medrxiv.org/content/10.1101/2021.10.26.21265397v1
